# Clinical evaluation of rituximab combined with ACE inhibitors and statins in refractory nephrotic syndrome: associations with proteinuria, lipid profiles, and podocyte-related biomarkers

**DOI:** 10.3389/fphar.2026.1851207

**Published:** 2026-07-06

**Authors:** Mengmeng Zhuang, Minguo Chen

**Affiliations:** 1 Department of Nephrology, Cangnan County People’s Hospital, Wenzhou, Zhejiang, China; 2 Cardiothoracic Surgery Department, Cangnan County People’s Hospital, Wenzhou, Zhejiang, China

**Keywords:** blood lipid profile, podocytes, proteinuria, refractory nephrotic syndrome, rituximab, statins

## Abstract

**Background:**

In refractory nephrotic syndrome (RNS) poorly responsive to standard therapy, combining rituximab (RTX) with Angiotensin-converting enzyme inhibitors (ACEI) and statins shows synergistic potential, yet its efficacy and podocyte effects require confirmation.

**Objective:**

To assess RTX plus ACEI and statin in RNS via propensity score matching (PSM), evaluating effects on proteinuria, lipids, and podocyte markers.

**Methods:**

Clinical data from RNS patients (January 2023–January 2025) were reviewed. The study compared a combination arm (RTX + ACEI + statin) versus a control arm (RTX + ACEI). PSM (1:1) adjusted for key baseline covariates. Assessments occurred at baseline, 3, 6, and 12 months. The 1-year primary endpoint was complete remission (protein <0.3 g/24 h, albumin >35 g/L). Secondary endpoints: partial remission, proteinuria decline, lipid/renal parameters, and podocyte markers. Confounders were addressed via multivariate logistic regression. Pearson correlation and multiple linear regression assessed associations between proteinuria improvement and changes in lipids or podocyte markers. Sensitivity analyses employed caliper widths of 0.05 and 0.15.

**Results:**

PSM yielded 28 patients per group, and all baseline factors were well balanced (SMD<0.1). At 12 months, the combination group outperformed controls in both complete remission (50.0% vs. 28.6%) and overall response (82.1% vs. 57.1%). This arm also showed sustained reductions in proteinuria alongside improved serum albumin throughout follow-up (P < 0.05). TC, TG, and LDL-C improved more in the combination group (P < 0.05), with no between-group difference in HDL-C Urinary podocyte markers declined more notably in the combination group and correlated with proteinuria reduction (r = 0.55–0.59, all P < 0.05). Complete remission was independently predicted by the combination group (OR = 3.33) and lower baseline proteinuria (OR = 0.78), whereas proteinuria improvement was tied to declines in podocalyxin (β = 0.32) and LDL-C (β = 2.15). Sensitivity analyses upheld the main results, with no group differences in safety profiles.

**Conclusion:**

In RNS, adding a statin to RTX–ACEI improved 12-month remission, proteinuria, lipids, and podocyte markers; prospective validation is needed.

## 1 Introduction

Refractory nephrotic syndrome (RNS) refers to a group of glomerular diseases that do not respond well to adequate glucocorticoid or combined immunosuppressive therapy, or may be remitted but relapse repeatedly. Patients with RNS are at risk of persistent massive proteinuria, progressive renal dysfunction, and cardiovascular events for a long time. Management remains challenging (Bianchi et al., 2025). Podocyte injury is the core pathological link in the occurrence and persistence of proteinuria, while nephrotic syndrome is often accompanied by severe lipid metabolism disorder, which can further aggravate renal injury by inducing oxidative stress of podocyte, promoting mesangial cell proliferation and glomerulosclerosis (Hackl and Weber, 2025; Tolerico et al., 2025). The dyslipidemia in nephrotic syndrome is primarily driven by two mechanisms: (i) hypoalbuminemia-induced compensatory hepatic overproduction of lipoproteins, including very-low-density lipoprotein (VLDL) and low-density lipoprotein (LDL); and (ii) reduced clearance of triglycerides and cholesterol due to decreased activity of lipoprotein lipase and other key enzymes in peripheral tissues. This results in elevated levels of total cholesterol, triglycerides, and LDL-C, which not only increase cardiovascular risk but also contribute directly to glomerular injury and podocyte dysfunction.

Rituximab (RTX), a B-cell-targeting monoclonal antibody, has gained widespread use for RNS in recent years (Aslam and Koirala, 2023). In addition to depleting B cells and inhibiting the production of autoantibodies, RTX can also directly protect the integrity of the cytoskeleton of podocytes by binding to SMPDL3b on the surface of podocytes (Fornoni et al., 2011; Zhao et al., 2025). Although RTX has shown good efficacy in some RNS patients, there are still a considerable proportion of patients who have a poor response to it (Jin et al., 2025). Angiotensin-converting enzyme inhibitors (ACEI) attenuate kidney function deterioration through reductions in intraglomerular pressure and proteinuria (Avula et al., 2025). Beyond lowering lipids, statins exert anti-inflammatory, anti-fibrotic, and direct podocyte-protective effects (Sadeghi et al., 2025). In theory, the multi-target treatment strategy of RTX combined with ACEI and statins may play a synergistic role through immune regulation, reducing intraglomerular pressure and improving lipid metabolism.

However, most of the existing evidence comes from the comparative studies of RTX and traditional immunosuppressants. For example, Huang et al. (Huang et al., 2025) recently found that RTX had a significantly better complete remission rate than cyclophosphamide and calcineurin inhibitors in membranous nephropathy. ACEI and statins may only be used as background therapy. As Zhu et al. (Zhu et al., 2025) have pointed out, RAS inhibitors and statins have been the basic treatment drugs for chronic kidney disease. So far, no study has directly evaluated the clinical value of adding statins to immunosuppression with “RTX plus ACEI” as a control. Moreover, the impact of combination therapy on podocyte markers and their relationship with proteinuria and lipid profiles remains unexplored.

In this context, this retrospective analysis applied propensity score matching to assess RTX plus ACEI with a statin in RNS, evaluating effects on proteinuria, lipids, and podocyte markers. The following hypothesis is proposed: The addition of statins to RTX combined with ACEI therapy may be associated with the improvement of complete remission rate in patients with RNS, accompanied by the improvement of proteinuria, lipid profile and podocyte injury indicators. By testing these hypotheses, this investigation intends to furnish real-world insights into combined therapies for RNS, with implications for clinical management.

## 2 Materials and methods

### 2.1 Study design

**FIGURE 1:**
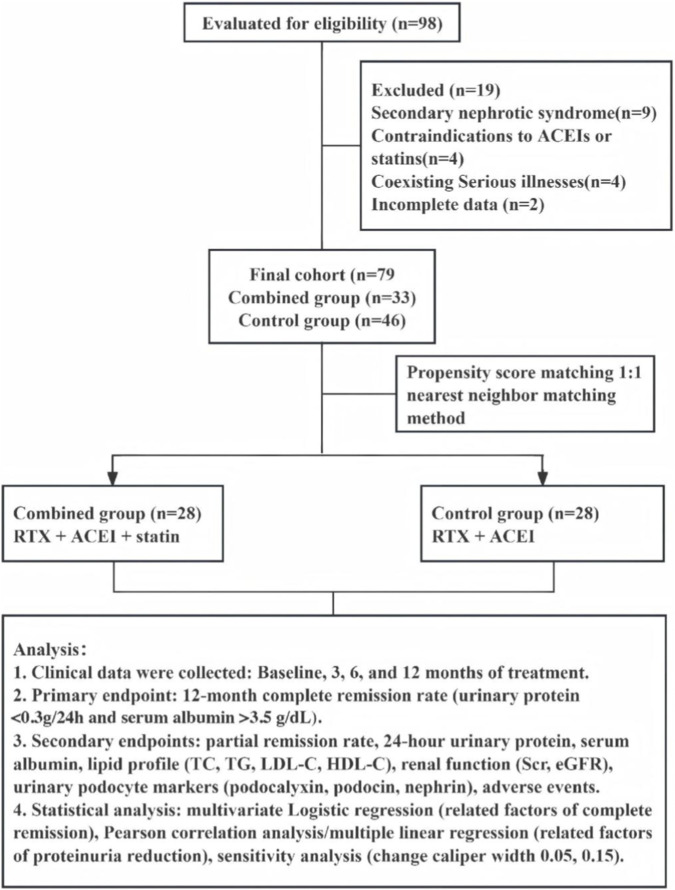
Design flow chart.

This retrospective cohort, conducted at a single center, included all RNS cases seen in our nephrology department between January 2023 and January 2025. Regimen-based grouping yielded two arms: combination (RTX + ACEI + statin) and control (RTX + ACEI). PSM was applied to assess the clinical efficacy of rituximab plus ACEI and statins in RNS, along with its impact on proteinuria, lipid profiles, and podocyte-related biomarkers. Approval was granted by the institutional ethics committee of Cangnan County People’s Hospital (Approval Number: 2025009). Patient consent was not required because the analysis used retrospective, anonymized data. The ethics committee also waived the requirement for informed consent for the use of residual biosamples for exploratory biomarker analysis, as all samples were anonymized and the study involved no more than minimal risk. The study followed the Declaration of Helsinki and relevant ethical guidelines (Association, 2025). Figure 1 outlines the overall study procedure.

### 2.2 Inclusion and exclusion criteria

Inclusion criteria: ① Age 18–75 years old; ② Renal biopsy-proven glomerular disease (minimal change disease, focal segmental glomerulosclerosis, or membranous nephropathy); ③ Meeting the KDIGO-based definition of refractory nephrotic syndrome (RNS) (Ravani et al., 2016) which includes: Steroid-resistant nephrotic syndrome (SRNS): no complete or partial remission after 8 weeks of full-dose prednisone (1 mg/kg/day or equivalent); Steroid-dependent nephrotic syndrome (SDNS): relapse within 2 weeks of steroid tapering or cessation, or two or more relapses within 6 months of initial response despite prior steroid sensitivity; Refractoriness to other immunosuppressants: failure to achieve remission after ≥12 weeks of adequate treatment with calcineurin inhibitors, cyclophosphamide, or mycophenolate mofetil, regardless of steroid status; ④ Patients had received ACEI treatment for more than 3 months before RTX treatment and had completed clinical follow-up for at least 12 months; ⑤ Complete clinical records.

Exclusion criteria: ① Secondary nephrotic syndrome (such as lupus nephritis, diabetic nephropathy, hepatitis B virus-related nephritis, amyloidosis, etc.); ② Contraindications to ACEI or statins use (such as bilateral renal artery stenosis, severe liver dysfunction, active liver disease); ③ previous kidney transplantation; ④ complicated with active infection, malignant tumor or severe heart, liver or lung dysfunction; ⑤ pregnant or lactating women.

### 2.3 Sample size calculation

The retrospective nature of the study informed sample size calculations, which were based on the 12-month complete remission endpoint. Based on literature reports, the complete remission rate of RNS patients treated with RTX is about 35%–40% (Zhao et al., 2025; Qi et al., 2026), and the CRR of the combination group is expected to be about 60%–65%. With a two-sided α of 0.05 and power of 80%, PASS 15.0 estimated that 26 patients per group were needed. Considering the possible sample size loss in the propensity score matching process, the total sample size was planned to be no less than 60. Finally, 79 patients who met the criteria were screened, and 56 patients (28 in each group) were matched to meet the sample size requirements.

### 2.4 Treatment methods

All treatment regimens were determined by clinical practice, not predefined by the study protocol, given the retrospective design. The treatment plan was individualized by the attending physician according to the patient’s condition, guideline recommendation and patient’s wishes.

#### 2.4.1 Rituximab regimen

All patients were treated with rituximab, according to medical records. The treatment plan was determined according to clinical routine and previous literature (Meeuwisse et al., 2022; Del Vecchio et al., 2021). Antihistamines and glucocorticoids were routinely used before the first dose to reduce the risk of infusion reactions. The regimen of rituximab was determined by the attending physician according to the patient’s body weight, body surface area and condition. The following two regimens were used: (Bianchi et al., 2025): Four weekly infusions of 375 mg/m^2^; (Hackl and Weber, 2025); Two 1-g doses spaced 2 weeks apart. During the treatment, the subsequent medication was adjusted according to the patient’s CD20^+^ B cell count and clinical response.

#### 2.4.2 ACEI treatment regimens

All enrolled patients were treated with ACEI and conformed to the standard adjuvant treatment regimen for nephrotic syndrome (Azukaitis et al., 2022). Enalapril or benazepril was the primary ACEI, initiated at 5–10 mg/d and adjusted based on blood pressure and tolerance, and the target systolic blood pressure is controlled below 130 mmHg (if tolerated) or adjusted to the maximum dose that patients can tolerate, in line with KDIGO 2021 guidelines (Foti et al., 2021). To facilitate comparison between groups, ACEI doses were uniformly converted to enalapril equivalent doses.

#### 2.4.3 Statin regimens

The combination group received statins alongside rituximab and ACEI, consistent with CKD lipid management guidelines (Kim et al., 2025). Atorvastatin or rosuvastatin were the main drugs. The initial dose was atorvastatin 10–20 mg/d or rosuvastatin 5–10 mg/d, and the dose was adjusted according to the blood lipid level and tolerance. The target low density lipoprotein cholesterol was controlled <2.6 mmol/L, which was in line with ACC/AHA guidelines (Wilson et al., 2019). In addition to lipid-lowering effects, statins may also directly protect the podocyte cytoskeleton and reduce proteinuria by inhibiting the RhoA/ROCK pathway.

#### 2.4.4 Other treatments

Both groups received antihypertensives (e.g., diuretics, calcium channel blockers) and symptomatic or supportive care as clinically indicated (Georgianos and Agarwal, 2023). The use of glucocorticoids and immunosuppressive agents was determined by the treating physician according to the patient’s condition and was adjusted for potential confounders in the statistical analysis.

### 2.5 Evaluation indicators

#### 2.5.1 Baseline indicators

Patient demographic characteristics (age, sex), disease duration, pathological type, systolic blood pressure, diastolic blood pressure, ACEI equivalent dose, RTX duration, and pre-treatment (baseline) clinical indicators were collected by reviewing the electronic medical record system, including: 24-h urine protein, serum albumin, serum creatinine (Scr), estimated glomerular filtration rate (eGFR), total cholesterol (TC), triglyceride (TG), low density lipoprotein cholesterol (LDL-C), high density lipoprotein cholesterol (HDL-C).

#### 2.5.2 Main outcome measures

Complete remission rate at 12 months: Complete remission was defined as 24 h proteinuria <0.3 g/24 h with serum albumin >3.5 g/dL, assessed at 12 months ± 2 weeks after the first RTX dose (Wendt et al., 2024).

#### 2.5.3 Secondary outcome measures

1. Partial remission rate: defined as urinary protein falling by ≥ 50% to <3.5 g/24 h, accompanied by serum albumin >3 g/dL.
2. 24-h urinary protein quantification: quantitative values at 3, 6, and 12 months of treatment and their changes from baseline.
3. Serum albumin: levels at 3, 6, and 12 months of treatment.
4. Blood lipid profile: Serum concentrations of TC, TG, LDL-C, and HDL-C recorded at 3-, 6-, and 12-month follow-up.
5. Renal function: Scr and eGFR levels at 3, 6, and 12 months after treatment.
6. Morning urine samples were collected from eligible patients at baseline and 12 months. All samples were immediately frozen at −80 °C after collection and measured uniformly in a single batch to avoid repeated freeze-thaw cycles. Urinary podocalyxin, podocin, and nephrin were quantified using enzyme-linked immunosorbent assay (ELISA) kits specific for human podocalyxin, podocin, and nephrin, respectively. All assays were performed according to the manufacturers’ instructions. In brief, 96-well plates pre-coated with capture antibodies were incubated with diluted urine samples (1:2 in assay buffer) and serial dilutions of standard proteins. After washing, a horseradish peroxidase-conjugated detection antibody was added, followed by a chromogenic substrate. The reaction was stopped with stop solution, and absorbance was measured at 450 nm using a microplate reader. Each sample was run in duplicate, and the mean value was used for analysis. For validation, standard curves were generated using the recombinant proteins provided in each kit; all curves showed R^2^ values >0.99. The intra-assay coefficient of variation (CV) was <8% for all three markers, and the inter-assay CV was <12%, as determined by repeated measurements of quality control samples. The limit of detection was as specified by the respective manufacturers. All urinary podocyte marker values were normalized to urinary creatinine concentration and expressed as μg/g Cr.
7. Safety indicators: Data on adverse events occurring throughout the treatment and follow-up period were obtained from electronic medical records and follow-up notes, including upper respiratory tract infection, infusion-related reactions, abnormal liver function, elevated creatine kinase, hypotension, etc. Adverse event severity was classified per CTCAE version 5.0 (Spampinato et al., 2025).

### 2.6 Statistical analysis

To minimize selection bias and confounding, 1:1 nearest-neighbor propensity score matching was applied. A logistic regression model estimated propensity scores using covariates: age, sex, baseline eGFR, 24-h urinary protein, serum albumin, pathological type (MCD/FSGS vs. membranous nephropathy), and RTX regimen (Gomez-Montero et al., 2025), with a caliper of 0.02. The standardized mean difference (SMD) served as the balance metric; values below 0.1 indicated well-matched covariates between arms. Continuous variables with normal distribution are expressed as mean ± SD; group differences were assessed by independent samples t-test. Non-normally distributed data are presented as median (IQR) and compared using the Mann-Whitney U test. Categorical variables are expressed as n (%); between-group differences were assessed by chi-square or Fisher’s exact test. Multivariate logistic regression assessed the association between combination therapy and complete remission, with treatment group as the primary independent variable, adjusted for age, baseline proteinuria, pathological type, baseline eGFR, and RTX course. Results are presented as odds ratios (OR) and corresponding 95% confidence intervals (CI). Pearson correlation analysis (when the data were in normal distribution) or Spearman correlation analysis (when the data were not normal distribution) was used to evaluate the relationship between the decrease of proteinuria and the changes of blood lipid (ΔTC, ΔTG, ΔLDL-C, ΔHDL-C) and podocyte markers (Δpodocalyxin, Δpodocin, Δnep) hrin). Multivariate linear regression analysis was used to adjust for potential confounding factors (treatment group, Δpodocalyxin, ΔLDL-C, baseline 24-h urinary protein, baseline eGFR (≥60 vs. < 60) and pathological type) to explore the independent influencing factors of the reduction of proteinuria. To test the robustness of the PSM results, the matching caliper width was changed to 0.05 and 0.15, and the PSM and primary endpoint analysis were repeated to observe the consistency of the results. The cases with missing key variables (primary endpoint and baseline data) were excluded. The last observation carried forward approach was used to impute missing data for secondary endpoints during follow-up. Data processing was carried out in SPSS 26.0 and R 4.3.1. A two-sided test threshold of α = 0.05 was applied, defining P < 0.05 as statistically significant.

## 3 Results

### 3.1 Baseline characteristics

From 79 screened patients, propensity score matching yielded 28 subjects in both the combination and control groups. After matching, the SMD of all covariates was less than 0.1, indicating that the baseline data of the two groups were well balanced. There were no significant differences in age, gender, course of disease, pathological type, blood pressure, ACEI equivalent dose, RTX course and laboratory indicators (24-h urinary protein quantification, serum albumin, eGFR, lipid profile) between the two groups (P > 0.05), which were comparable (Table 1). Regarding rituximab regimens, among the 56 matched patients, 34 received the 4-weekly 375 mg/m^2^ regimen (18 in the combination group, 16 in the control group) and 22 received the two-dose 1 g regimen (10 in the combination group, 12 in the control group). The complete remission rate at 12 months was comparable between the two regimens (47.1% vs. 45.5%, P = 0.91), and no significant interaction with treatment group was observed.

**TABLE 1:** Baseline characteristics of patients after matching.

Variable	Combined group (n = 28)	Control group (n = 28)	Statistic	P-value	SMD
Age (years, x̄±s)	41.3 ± 12.5	42.1 ± 13.2	t = −0.241	0.81	0.062
Male [n (%)]	16 (57.1)	15 (53.6)	χ^2^ = 0.071	0.79	0.071
Disease duration (months, median [IQR])	18.5 (12.0, 30.0)	20.0 (14.0, 32.0)	Z = −0.512	0.608	0.085
Pathological type [n (%)]	​	​	χ^2^ = 1.246	0.536	-
MCD	10 (35.7)	8 (28.6)	​	​	​
FSGS	2 (7.1)	6 (21.4)	​	​	​
Membranous nephropathy	16 (57.1)	14 (50.0)	​	​	​
Systolic BP (mmHg, x̄±s)	128.5 ± 10.2	130.1 ± 11.4	t = −0.542	0.59	0.069
Diastolic BP (mmHg, x̄±s)	83.2 ± 8.6	83.6 ± 8.8	t = −0.174	0.863	0.046
ACEI equivalent dose (mg/d, x̄±s)	12.5 ± 3.2	13.0 ± 3.5	t = −0.537	0.593	0.071
RTX course (weeks, median [IQR])	4.0 (4.0, 8.0)	4.0 (4.0, 8.0)	Z = 0.000	1.000	0.000
24 h urinary protein (g/24 h, x̄±s)	5.2 ± 1.5	5.5 ± 1.6	t = −0.720	0.475	0.089
Serum albumin (g/dL, x̄±s)	2.43 ± 0.45	2.38 ± 0.49	t = 0.382	0.704	0.064
eGFR (mL/min/1.73 m^2^, x̄±s)	68.5 ± 15.2	66.9 ± 16.5	t = 0.366	0.716	0.063
TC (mg/dL)	263.7 ± 52.2	267.2 ± 54.9	t = −0.245	0.807	0.058
TG (mg/dL)	217.0 ± 75.3	223.2 ± 79.7	t = −0.295	0.769	0.074
LDL-C (mg/dL)	159.3 ± 41.8	162.4 ± 44.5	t = −0.271	0.787	0.060
HDL-C (mg/dL)	41.8 ± 9.7	40.6 ± 10.8	t = 0.426	0.672	0.069

MCD, minimal change disease; FSGS, focal segmental glomerulosclerosis; eGFR, estimated glomerular filtration rate; TC, total cholesterol; TG, triglycerides; LDL-C, low-density lipoprotein cholesterol; HDL-C, high-density lipoprotein cholesterol; SMD, standardized mean difference.

### 3.2 12 months complete remission rate

At 12 months, the combination group achieved a significantly higher complete remission rate than controls (50.0% vs. 28.6%, χ^2^ = 3.077, P = 0.040, OR = 3.33, 95%CI 1.16–9.52). The overall remission rate was also significantly higher in the combination group versus controls (82.1% vs. 57.1%, χ^2^ = 4.050, P = 0.044; OR = 3.45, 95% CI 1.06–11.20). No significant between-group difference was observed in partial response (32.1% vs. 28.6%, P = 0.775). The combination group had a significantly reduced non-response rate relative to controls (17.9% vs. 42.9%, P = 0.049) (Table 2). In addition, we explored the 12-month complete remission rates by pathological subtype. For MCD (n = 18: 10 in combination group, 8 in control), the CR rate was 70.0% (7/10) in the combination group vs. 37.5% (3/8) in controls; for FSGS (n = 8: 2 vs. 6), 50.0% (1/2) vs. 16.7% (1/6); for MN (n = 30: 16 vs. 14), 37.5% (6/16) vs. 28.6% (4/14). Despite the small sample sizes, the combination therapy showed numerically higher CR rates across all subtypes.

**TABLE 2:** Remission outcomes at 12 months by group [n (%)].

Indicator	Combined group (n = 28)	Control group (n = 28)	χ^2^-value	P-value
Complete remission	14 (50.0)	8 (28.6)	3.077	0.040
Partial remission	9 (32.1)	8 (28.6)	0.082	0.775
No remission	5 (17.9)	12 (42.9)	3.889	0.049
Overall remission	23 (82.1)	16 (57.1)	4.050	0.044

Remission was defined as follows: complete–protein <0.3 g/24 h and albumin >35 g/L; partial – ≥ 50% drop in proteinuria to <3.5 g/24 h and albumin >30 g/L (complete remitters excluded). Overall remission included both categories.

To further examine whether the improvements in lipid profiles and podocyte markers were confined to patients who achieved complete remission, we performed a stratified analysis within the combination group. As shown in Supplementary Table S1, patients who achieved CR (n = 14) had significantly greater reductions in LDL-C (Δ −2.13 ± 0.76 mmol/L vs. −1.31 ± 0.58 mmol/L, P = 0.002) and podocalyxin (Δ −9.2 ± 3.1 μg/g Cr vs. −5.1 ± 2.9 μg/g Cr, P < 0.001) compared with non-CR patients (n = 14). The increase in HDL-C did not differ significantly between the two subgroups (Δ +0.41 ± 0.22 mmol/L vs. +0.33 ± 0.24 mmol/L, P = 0.29). These findings suggest that the benefits of adding a statin on LDL-C reduction and podocyte protection are more pronounced in patients who attain remission. Due to the small sample size and exploratory nature, these results should be interpreted with caution.

### 3.3 Changes in renal function, proteinuria and serum albumin

**FIGURE 2:**
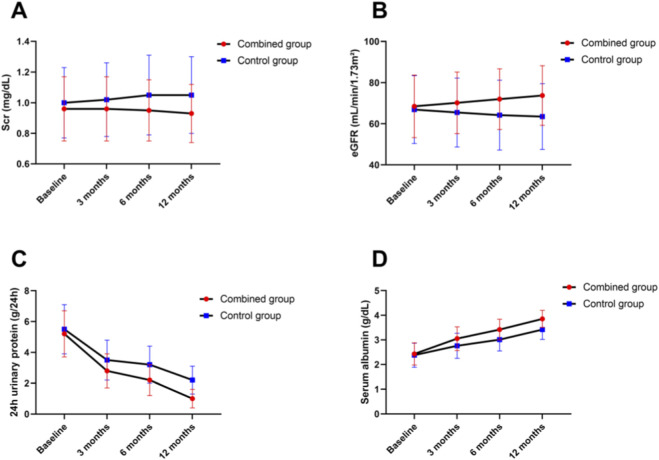
Temporal changes in renal function, proteinuria, and serum albumin. Line plots showing **(A)** serum creatinine (mg/dL) **(B)** estimated glomerular filtration rate (eGFR, mL/min/1.73 m^2^) **(C)** 24-h urinary protein (g/24 h), and **(D)** serum albumin (g/dL) over 12 months. Data are presented as mean ± SD. Red circles represents the combination group (RTX + ACEI + statin); blue squares represents the control group (RTX + ACEI). P values at each time point are provided in Table 3. Abbreviations: RTX, rituximab; ACEI, angiotensin-converting enzyme inhibitor.

Baseline values for Scr, eGFR, 24-h urinary protein, and serum albumin did not differ significantly between groups (P > 0.05). At 12 months, the combination group had significantly lower Scr and higher eGFR than controls (P = 0.033 and P = 0.011, respectively). At 3, 6, and 12 months, the combination group showed significantly lower proteinuria and higher serum albumin than controls (P < 0.05 for all) (Figure 2; Table 3).

**TABLE 3:** Renal function, proteinuria, and serum albumin over time (x̄±s).

Indicator	Combined group (n = 28)	Control group (n = 28)	t-value	P-value
Scr (mg/dL)				
Baseline	0.96 ± 0.21	1.00 ± 0.23	−0.658	0.514
3 months	0.96 ± 0.21	1.02 ± 0.24	−1.082	0.284
6 months	0.95 ± 0.20	1.05 ± 0.26	−1.623	0.110
12 months	0.93 ± 0.19	1.05 ± 0.25	−2.184	0.033
eGFR (mL/min/1.73 m^2^)				
Baseline	68.5 ± 15.2	66.9 ± 16.5	0.366	0.716
3 months	70.2 ± 15.0	65.5 ± 16.8	1.101	0.276
6 months	72.0 ± 14.8	64.2 ± 17.0	1.843	0.071
12 months	73.8 ± 14.5	63.5 ± 16.0	2.641	0.011
24 h urinary protein (g/24 h)				
Baseline	5.2 ± 1.5	5.5 ± 1.6	−0.72	0.475
3 months	2.8 ± 1.1	3.5 ± 1.3	−2.214	0.031
6 months	2.2 ± 1.0	3.2 ± 1.2	−3.507	0.003
12 months	1.0 ± 0.6	2.2 ± 0.9	−5.599	<0.001
Serum albumin (g/dL)				
Baseline	2.43 ± 0.45	2.38 ± 0.49	0.382	0.704
3 months	3.05 ± 0.48	2.76 ± 0.51	2.19	0.033
6 months	3.42 ± 0.42	3.01 ± 0.46	3.463	0.001
12 months	3.85 ± 0.35	3.42 ± 0.40	4.308	<0.001

Mean ± SD, is reported for continuous variables, and groups were compared using the independent samples t-test.

### 3.4 Changes in blood lipid profile

**FIGURE 3:**
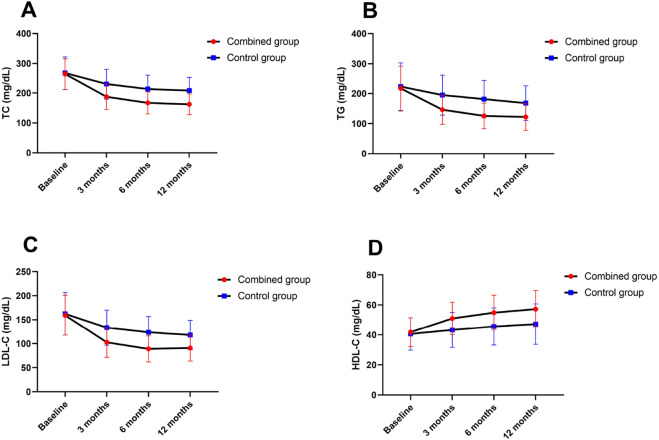
Temporal changes in lipid profiles. Line plots showing **(A)** total cholesterol (TC, mg/dL) **(B)** triglycerides (TG, mg/dL) **(C)** low-density lipoprotein cholesterol (LDL-C, mg/dL), and **(D)** high-density lipoprotein cholesterol (HDL-C, mg/dL) over 12 months. Data are presented as mean ± SD. Red circles represents the combination group (RTX + ACEI + statin); blue squares represents the control group (RTX + ACEI). P values at each time point are provided in Table 4. Abbreviations: RTX, rituximab; ACEI, angiotensin-converting enzyme inhibitor.

Baseline TC, TG, LDL-C, and HDL-C levels were comparable between groups (P > 0.05). At 3, 6, and 12 months, the combination group showed significantly lower TC, TG, and LDL-C and higher HDL-C than controls (P < 0.05 for all) (Figure 3; Table 4).

**TABLE 4:** Serial lipid profile changes by group (x̄±s).

Indicator	Combined group (n = 28)	Control group (n = 28)	t-value	P-value
TC (mg/dL)				
Baseline	263.7 ± 52.2	267.2 ± 54.9	−0.245	0.807
3 months	187.5 ± 42.5	230.1 ± 50.3	−3.528	0.001
6 months	167.1 ± 36.7	213.5 ± 46.4	−4.183	<0.001
12 months	162.4 ± 34.8	208.1 ± 44.5	−4.930	<0.001
TG (mg/dL)				
Baseline	217.0 ± 75.3	223.2 ± 79.7	−0.295	0.769
3 months	146.1 ± 48.7	194.9 ± 66.4	−3.156	0.003
6 months	125.8 ± 42.5	181.6 ± 62.0	−3.995	<0.001
12 months	122.2 ± 44.3	168.3 ± 57.6	−4.000	<0.001
LDL-C (mg/dL)				
Baseline	159.3 ± 41.8	162.4 ± 44.5	−0.271	0.787
3 months	102.5 ± 30.9	133.4 ± 36.7	−3.466	0.001
6 months	89.0 ± 27.1	123.7 ± 32.9	−4.326	<0.001
12 months	90.9 ± 27.1	118.0 ± 30.9	−4.637	<0.001
HDL-C (mg/dL)				
Baseline	41.8 ± 9.7	40.6 ± 10.8	0.426	0.672
3 months	51.0 ± 10.8	43.3 ± 11.6	2.597	0.012
6 months	54.9 ± 11.6	45.6 ± 12.4	2.893	0.006
12 months	57.2 ± 12.4	47.2 ± 13.5	3.147	0.003

Values are mean ± SD; groups were compared by independent t-test.

### 3.5 Urinary podocyte markers

**FIGURE 4:**
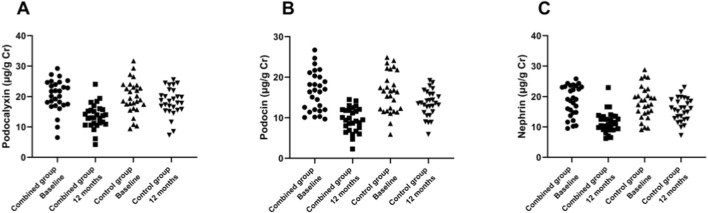
Urinary podocyte markers at baseline and 12 months. Boxplots with scatterplots of **(A)** podocalyxin **(B)** podocin, and **(C)** nephrin. Each dot represents one patient. P < 0.001 for all three markers at 12 months (combination vs. control). Data are in μg/g Cr.

At baseline, urinary podocalyxin, podocin, and nephrin levels were comparable between groups (P > 0.05). The combination arm showed significantly reduced levels of podocalyxin, podocin, and nephrin at 12 months compared with the control group (P < 0.001) (Table 5). As shown in Figure 4, the distribution of individual values confirms this improvement, with most patients in the combination group achieving lower podocyte marker levels at 12 months.

**TABLE 5:** Urinary podocyte markers pre- and post-treatment by group (x̄±s).

Indicator	Combined group (n = 28)	Control group (n = 28)	t-value	P-value
Podocalyxin (μg/g cr)				
Baseline	20.3 ± 5.2	19.8 ± 5.5	0.347	0.73
12 months	13.5 ± 4.0	18.2 ± 4.5	−5.087	<0.001
Podocin (μg/g cr)				
Baseline	16.5 ± 4.8	16.2 ± 5.0	0.228	0.821
12 months	9.5 ± 3.0	13.5 ± 3.2	−6.654	<0.001
Nephrin (μg/g cr)				
Baseline	18.6 ± 5.0	18.1 ± 5.3	0.363	0.718
12 months	11.5 ± 3.5	15.6 ± 3.8	−5.944	<0.001

Mean ± SD, reported; groups compared using the independent t-test.

### 3.6 Multivariate logistic regression analysis

Multivariate logistic regression examined complete remission at 12 months, with treatment group, age, baseline proteinuria, baseline eGFR, pathology, and prior immunosuppression as covariates. Combination therapy (OR = 3.33, 95% CI 1.16–9.52, P = 0.032) and lower baseline proteinuria (OR = 0.78, 95% CI 0.62–0.98, P = 0.032) emerged as independent predictors of complete remission. Baseline eGFR (OR = 1.20, 95%CI 0.97–1.49, P = 0.091), pathological type, and previous immunosuppressive therapy did not reach statistical significance for complete response (Table 6).

**TABLE 6:** Multivariate logistic regression: predictors of complete remission.

Variable	β coefficient	Wald χ^2^	Or (95%CI)	P-value
Combination therapy (vs. control group)	1.204	5.982	3.33 (1.16–9.52)	0.032
Baseline 24 h urinary protein (per 1 g increase)	−0.248	4.62	0.78 (0.62–0.98)	0.032
Baseline eGFR (per 10 mL/min/1.73 m^2^ increase)	0.185	2.856	1.20 (0.97–1.49)	0.091
Pathological type (MCD/FSGS vs. MN)	0.685	2.105	1.98 (0.78–5.02)	0.147
Prior immunosuppressive therapy (yes vs. no)	−0.512	1.642	0.60 (0.27–1.34)	0.21
Age (per 1 year increase)	−0.021	1.024	0.98 (0.94–1.02)	0.312

The outcome variable was complete remission status at 12 months (1 = achieved, 0 = not). Prior immunosuppressive treatment included cyclophosphamide, calcineurin inhibitors, or mycophenolate mofetil.

### 3.7 Correlation analysis

Pearson correlation analysis showed that the reduction of proteinuria at 12 months after treatment was positively correlated with ΔTC, ΔTG, ΔLDL-C and three podocyte markers (ΔPodocalyxin, ΔPodocin and ΔNephrin, r = 0.45–0.63, P < 0.01). Among them, ΔPodocin had the strongest correlation (r = 0.63), suggesting that the improvement of podocyte slit membrane protein was most closely related to the reduction of proteinuria. ΔHDL-C (r = 0.18, P = 0.182) and ΔeGFR (r = 0.24, P = 0.068) did not reach statistical significance in relation to the reduction in proteinuria, which may be related to the delayed improvement in renal function (Table 7).

**TABLE 7:** Correlation between the magnitude of proteinuria reduction and changes in various indicators.

Variable	Correlation coefficient (r)	P-value
Lipid changes(mg/dL)		
ΔTC	0.48	0.002
ΔTG	0.52	<0.001
ΔLDL-C	0.45	0.004
ΔHDL-C	0.18	0.182
Renal function changes (mL/min/1.73 m^2^)		
ΔeGFR	0.24	0.068
Podocyte marker changes (μg/g cr)		
ΔPodocalyxin	0.59	<0.01
ΔPodocin	0.63	<0.01
ΔNephrin	0.55	<0.01

Δ denotes the change from baseline to 12 months (12-month value–baseline). Reduction in proteinuria = baseline 24 h urinary protein – 12-month value. Correlations were assessed by Pearson’s test, uncorrected for multiplicity.

### 3.8 Multiple linear regression analysis

Multiple linear regression was performed with proteinuria reduction as the outcome, incorporating treatment group, ΔPodocalyxin, ΔLDL-C, baseline 24 h protein, baseline eGFR (≥60 vs. <60), and pathological type. Independent positive predictors of proteinuria reduction were combination therapy (β = 4.85), ΔPodocalyxin (β = 0.32), ΔLDL-C (β = 0.056), and baseline eGFR ≥60 (β = 3.25) (all P < 0.05), while baseline proteinuria (β = −1.20) was a negative predictor (P = 0.042). The model-adjusted R^2^ = 0.48 indicated that the included variables explained 48% of the variation in the reduction in proteinuria (Table 8).

**TABLE 8:** Independent factors associated with proteinuria decline (multiple linear regression).

Variable	β coefficient	Standard error	Standardized β	P-value	95% CI
Group (combination = 1, control = 0)	4.85	1.52	0.32	0.002	1.81–7.89
ΔPodocalyxin (μg/g cr)	0.32	0.09	0.38	0.001	0.14–0.50
ΔLDL-C (mg/dL)	0.056	0.022	0.24	0.015	0.012–0.100
Baseline 24 h urinary protein (g/24 h)	−1.20	0.58	−0.18	0.042	−2.36–−0.04
Baseline eGFR ≥60 (yes = 1, no = 0)	3.25	1.45	0.22	0.028	0.35–6.15
Pathological type (MN = 1, non-MN = 0)	−2.10	1.80	−0.12	0.248	−5.70–1.50

The outcome variable was proteinuria reduction (g/24 h) = baseline minus 12-month urinary protein. The model showed adjusted R^2^ = 0.48, F = 8.65, P < 0.001. Δ represents the 12-month value minus baseline.

### 3.9 Safety analysis

The frequency of adverse events did not differ significantly across groups (P > 0.05). The most frequent adverse events were infusion-related reactions (17.9% vs. 14.3%) and upper respiratory tract infections (14.3% vs. 10.7%), all mild to moderate (CTCAE grade 1–2) and resolved with symptomatic care. No serious adverse events or deaths were reported. See Table 9 for details.

**TABLE 9:** Adverse event rates by group [n (%)].

Adverse event	Combined group (n = 28)	Control group (n = 28)	χ^2^ value	P-value
Infusion-related reaction	5 (17.9)	4 (14.3)	0.131	0.717
Upper respiratory tract infection	4 (14.3)	3 (10.7)	0.161	0.688
Hepatic dysfunction	2 (7.1)	1 (3.6)	-	1.000
Hypotension	1 (3.6)	2 (7.1)	-	1.000
Elevated creatine kinase	1 (3.6)	0 (0.0)	-	1.000
Any adverse event	11 (39.3)	8 (28.6)	0.700	0.403

Toxicity was graded using CTCAE, version 5.0, with all events being grade 1 or 2. The chi-square test was applied when expected frequencies reached ≥5; Fisher’s exact test was used otherwise.

### 3.10 Sensitivity analysis

In order to verify the robustness of the propensity score matching results, the matching caliper width was adjusted to 0.05 and 0.15, respectively, and then the matching was performed again. Sensitivity analyses with calipers set to 0.05 and 0.15 yielded results consistent with the primary analysis: complete remission rates favored the combination group (58.3% vs. 29.2%, P = 0.037; and 57.7% vs. 30.8%, P = 0.042, respectively). Odds ratios and 95% confidence intervals aligned with the primary analysis, indicating robust findings (Table 10).

**TABLE 10:** Sensitivity analysis of the primary outcome.

Analysis approach	Matched n per group	CRR in combined group (%)	CRR in control group (%)	P-value	Or (95% CI)
Primary analysis (PSM, caliper = 0.02)	28	50.0 (14/28)	28.6 (8/28)	0.040	3.33 (1.16–9.52)
Sensitivity analysis 1 (PSM, caliper = 0.05)	24	58.3 (14/24)	29.2 (7/24)	0.037	3.42 (1.12–10.45)
Sensitivity analysis 2 (PSM, caliper = 0.15)	26	57.7 (15/26)	30.8 (8/26)	0.042	3.08 (1.03–9.21)

Complete remission rate (CRR) was defined by protein <0.3 g/24 h plus albumin >35 g/L. Between-group differences were assessed via χ^2^ test. The OR, indicates the odds of remission for the combination group relative to controls.

## 4 Discussion

This study retrospectively evaluated the efficacy of rituximab plus ACEI and statins in RNS using PSM. Adding a statin to RTX plus ACEI was associated with a higher 12-month complete remission rate (50.0% vs. 28.6%, OR = 3.33), as well as improvements in proteinuria, lipid profile and podocyte injury indicators. Sensitivity analyses confirmed the robustness of the findings, and safety analysis revealed no increased risk of adverse events with the triple regimen.

The 12-month complete remission rate in the control group (RTX + ACEI) was 28.6%, aligning with previous reports of RTX efficacy in RNS. Xu et al. (Xu et al., 2022) showed that the overall remission rate of RTX treatment was 65%, and the complete remission rate was about 33% in the prospective study of RNS patients, and the results of the control group in this study were similar. In another classic study, Ruggenenti et al. (Ruggenenti et al., 2014) reported that the complete remission rate of patients treated with RTX combined with ACEI and statins was 25%, which further supported the stability of the basic efficacy of RTX combined with ACEI. The combination group achieved a higher complete remission rate (50.0%) than previously reported, a difference potentially attributable to multiple factors. On the one hand, statins in the triple therapy may act synergistically with RTX through their pleiotropic effects—lowering lipids, reducing inflammation, and directly protecting podocytes. On the other hand, MCD/FSGS accounted for 64.3% of the patients in this study, and this pathological type usually responds better to RTX than membranous nephropathy. In addition, the between-group difference in the rate of complete response in our study became apparent at 6 months and further increased by 12 months, which suggests that the synergistic effect of statins may take time to accumulate, possibly related to improved lipid metabolism and delayed podocyte repair. This time trend was consistent with the rule reported by Feder et al. (Feder et al., 2025) that the time to recurrence after RTX treatment was significantly prolonged (median time to first recurrence increased from 11 days before treatment to 536 days after treatment, P = 0.002).

Based on the efficacy analysis, the effect of combined treatment on proteinuria, blood lipid profile and podocyte markers was further investigated. Parallel to the complete remission results, the triple-therapy group achieved superior proteinuria reduction and serum albumin improvement. In terms of lipid profile, TC, TG and LDL-C in the combined group decreased more significantly, which was consistent with the lipid-lowering effect of statins (Karami et al., 2024). The combination group also showed a modest but statistically significant increase in HDL-C at 3, 6, and 12 months compared with controls. This aligns with the modest effect of statins on HDL-C and may also reflect RTX-mediated lipid improvements (Liaig et al., 2025; Zhong et al., 2023). Changes in podocyte markers are one of the concerns of this study. At 12 months, urinary podocalyxin, podocin, and nephrin levels were lower in the combination group than in controls and correlated positively with proteinuria reduction (r = 0.55–0.63). Among them, podocin has the strongest correlation (r = 0.63). As the core protein of slit diaphragm, its improvement may reflect the repair of podocyte structure to a certain extent (Qadri et al., 2025; Fukuda et al., 2024). This observation echoes the mechanism proposed by Takahashi et al. (Takahashi et al., 2024) that RTX protects the cytoskeleton by binding to podocyte SMPDL3b, and is also consistent with the theory of Burke et al. (Burke et al., 2023) that RTX directly targets podocyte. In addition, statins can protect podocytes by inhibiting Rho kinase and reducing oxidative stress, and the improvement of podocyte markers observed in this study may reflect the synergistic effect of RTX and statins in podocyte protection (Ma et al., 2024). Multivariate analysis showed that ΔPodocalyxin and ΔLDL-C were independently associated with the decrease of proteinuria. This result suggests that podocyte protection and improvement of lipid metabolism may be two pathways through which combined therapy exerts its effects, and they may interact with each other to participate in the repair process of glomerular injury (Naik et al., 2026; Hu et al., 2024).

Based on these improvements in proteinuria and podocyte markers, the current study further examined the effect of combination therapy on renal function. At 12 months, eGFR increased modestly from baseline in the combination group but decreased slightly in controls, resulting in a significant between-group difference. The significant difference in eGFR occurred later than the improvement in proteinuria (between-group difference at 3 months), aligning with findings from recent publications. Varga-brochero et al. (Vargas-Brochero et al., 2025) conducted a long-term follow-up of 159 patients with membranous nephropathy and found that the median time to achieve partial remission after RTX treatment was 6.8 months, while the median time to achieve complete remission was as long as 22.6 months, indicating that there was a significant time lag between the improvement of renal function and the reduction of proteinuria. From a practical perspective, a stable or modest increase in eGFR in the combination-therapy group may suggest that the additive effect of statins may help to delay the decline in renal function, but extended observation is necessary to confirm this inference.

Along with efficacy, the safety of the combination approach was examined. Adverse event rates did not differ significantly between groups. The most frequently reported adverse events were infusion-related reactions (combined group 17.9%, control group 14.3%) and upper respiratory tract infection (combined group 14.3%, control group 10.7%), all of which were mild to moderate and could be relieved after symptomatic treatment. The combination group experienced no statin-associated severe hepatic events or notable increases in creatine kinase (>5 × ULN). Infusion reaction rates in this study aligned with the meta-analysis findings reported by Yu et al. (Yu et al., 2025), a retrospective study reported a 8% (95% CI 3%–13%) incidence of infusion reactions in nephrotic syndrome patients treated with RTX. Infusion reaction rates in our study were modestly higher than those reported in the meta-analysis, which may be related to the differences in the definition of infusion reactions, conditioning regimens and study populations. No elevated infection risk was noted in the combination group, potentially owing to the small sample size or limited follow-up duration. Overall, adding a statin to RTX plus ACEI had a good short-term safety profile.

In summary, the clinical significance of this study is mainly in the following aspects: first, using RTX combined with ACEI as a control, we tried to evaluate the clinical value of adding statins on the basis of immunosuppression, and to provide a preliminary observation on the potential role of statins in the combination regimen; Secondly, the association of podocyte markers with proteinuria and blood lipid profile was systematically analyzed to provide possible molecular clues for the synergistic mechanism of combined treatment. Third, PSM combined with sensitivity analysis was used to control confounding bias in the retrospective study. Fourth, to provide preliminary evidence for localization in this field based on real-world data of the Chinese population.

## 5 Limitations

Certain limitations warrant attention when evaluating this study’s conclusions. First, the retrospective design cannot completely rule out selection bias and residual confounding. Despite PSM and multivariate adjustment, unmeasured confounders (e.g., patient adherence, diet control, lifestyle) may still exist. Second, the sample size was relatively limited (28 cases in each group after matching), which may affect the statistical power of the subgroup analysis, especially for the membranous nephropathy subgroup (8–10 cases in each group). Third, the single-center design may limit the generalizability of the findings. The differences in RTX dosing regimens, ACEIs and statin dosage adjustment strategies among different centers may affect the reproducibility of the results. Fourth, with only 12 months of follow-up, long-term outcomes (e.g., recurrence, end-stage renal disease) and extended safety require further investigation. Fifth, this study did not perform stratified analysis of different pathological types (such as MCD/FSGS and membranous nephropathy), and whether there is a difference in the efficacy of statins in different pathological subgroups needs to be explored in future studies. Given these findings and limitations, future studies should consider the following directions: ① To evaluate the long-term renal function protection and recurrence risk by extending the follow-up time; ② To explore the optimal dose, type and initiation time of statins to optimize the combination regimen; ③ To explore the potential mechanism of combined therapy through more podocyte-related markers and imaging methods; ④ To carry out subgroup analysis of different pathological types to provide a basis for precise stratified treatment; ⑤ Incorporate additional cardiovascular endpoints to assess the impact of combination therapy on cardiovascular events in RNS patients.

## 6 Conclusion

In summary, adding a statin to RTX plus ACEI was associated with a higher 12-month complete remission rate in RNS patients, as well as improvements in proteinuria, lipid profile, and podocyte injury markers, with a favorable short-term safety profile. This combined strategy may provide an optimal treatment option for RNS, though its durability and safety warrant confirmation through prospective studies.

## Data Availability

The original contributions presented in the study are included in the article/Supplementary Material, further inquiries can be directed to the corresponding author.

## References

[B1] Aslam A. Koirala A. (2023). Review of the role of rituximab in the management of adult minimal change disease and immune-mediated focal and segmental glomerulosclerosis. Glomerular Dis. 3, 211–219. 10.1159/000533695 37901702 PMC10601923

[B2] Association W. M. (2025). World medical association declaration of helsinki: ethical principles for medical research involving human participants. Jama 333, 71–74. 10.1001/jama.2024.21972 39425955

[B3] Avula A. Johal L. K. Ali F. Amir S. Yadav S. Murtuza M. (2025). ACE inhibitors and ARBs in chronic kidney disease: a systematic review of randomized controlled trials on albuminuria reduction, eGFR decline, and safety. Cureus 17, e93707. 10.7759/cureus.93707 41189867 PMC12580586

[B4] Azukaitis K. Palmer S. C. Strippoli G. F. Hodson E. M. (2022). Interventions for minimal change disease in adults with nephrotic syndrome. Cochrane Database Syst. Rev. 3, Cd001537. 10.1002/14651858.CD001537.pub5 35230699 PMC8887628

[B5] Bianchi G. Bellucci L. Morello W. Turolo S. Cricrì G. Caicci F. (2025). Effects of steroid-resistant nephrotic syndrome serum on AA pathway in podocytes cultured in 3D in vitro glomerular model. Sci. Rep. 15, 12802. 10.1038/s41598-025-95216-2 40229314 PMC11997139

[B6] Burke G. W. 3rd Mitrofanova A. Fontanella A. Ciancio G. Roth D. Ruiz P. (2023). The podocyte: glomerular sentinel at the crossroads of innate and adaptive immunity. Front. Immunol. 14, 1201619. 10.3389/fimmu.2023.1201619 37564655 PMC10410139

[B7] Del Vecchio L. Allinovi M. Rocco P. Brando B. (2021). Rituximab therapy for adults with nephrotic syndromes: Standard schedules or B cell-targeted therapy? J. Clin. Med. 10, 5847. 10.3390/jcm10245847 34945143 PMC8709396

[B8] Feder O. Amsterdam D. Ershed M. Grupper A. Schwartz D. Kliuk-Ben Bassat O. (2025). Long-term efficacy of rituximab in steroid dependent and frequent relapsing adult nephrotic syndrome. BMC Nephrol. 26, 126. 10.1186/s12882-025-04035-0 40050772 PMC11887153

[B9] Fornoni A. Sageshima J. Wei C. Merscher-Gomez S. Aguillon-Prada R. Jauregui A. N. (2011). Rituximab targets podocytes in recurrent focal segmental glomerulosclerosis. Sci. Transl. Med. 3, 85ra46. 10.1126/scitranslmed.3002231 21632984 PMC3719858

[B10] Foti K. E. Wang D. Chang A. R. Selvin E. Sarnak M. J. Chang T. I. (2021). Potential implications of the 2021 KDIGO blood pressure guideline for adults with chronic kidney disease in the United States. Kidney Int. 99, 686–695. 10.1016/j.kint.2020.12.019 33637204 PMC7958922

[B11] Fukuda A. Sato Y. Shibata H. Fujimoto S. Wiggins R. C. (2024). Urinary podocyte markers of disease activity, therapeutic efficacy, and long-term outcomes in acute and chronic kidney diseases. Clin. Exp. Nephrol. 28, 496–504. 10.1007/s10157-024-02465-y 38402504 PMC11116200

[B12] Georgianos P. I. Agarwal R. (2023). Hypertension in chronic kidney disease-treatment standard 2023. Nephrol. Dial. Transpl. 38, 2694–2703. 10.1093/ndt/gfad118 PMC10689140 37355779

[B13] Gomez-Montero A. Garcia-Lopez A. Cabas S. Nieves-Rico A. A. Giron-Luque F. (2025). Methodological guidance on implementing propensity score matching in observational studies of kidney transplantation. Sci. Rep. 16, 1878. 10.1038/s41598-025-31596-9 41390565 PMC12804877

[B14] Hackl A. Weber L. T. (2025). The Ca(2+)-actin-cytoskeleton axis in podocytes is an important, non-immunologic target of immunosuppressive therapy in proteinuric kidney diseases. Pediatr. Nephrol. 40, 2729–2739. 10.1007/s00467-025-06670-z 39856247 PMC12296978

[B15] Hu J. Zhu Z. Zhang Z. Hu H. Yang Q. (2024). Blockade of STARD3-mediated cholesterol transport alleviates diabetes-induced podocyte injury by reducing mitochondrial cholesterol accumulation. Life Sci. 349, 122722. 10.1016/j.lfs.2024.122722 38754814

[B16] Huang J. Yang Y. Wang Y. Jiang S. Zhang Y. Zhao S. (2025). Effectiveness and safety of rituximab monotherapy versus conventional regimens for adult idiopathic membranous nephropathy: real-world retrospective study. Front. Immunol. 16, 1671251. 10.3389/fimmu.2025.1671251 41306981 PMC12643969

[B17] Jin Y. Xie Y. Fu H. Liu F. Mao J. (2025). Anti-B cell strategy in nephrotic syndrome: beyond rituximab. Biomedicines 13, 2063. 10.3390/biomedicines13092063 41007627 PMC12467319

[B18] Karami J. Razi B. Imani D. Aslani S. Pakjoo M. Fasihi M. (2024). Statin therapy and lipid indices in chronic kidney disease: a systematic review and meta-analysis of randomized control trials. Curr. Pharm. Des. 30, 362–376. 10.2174/0113816128285148240122112045 38288799

[B19] Kim J. Y. Chung S. M. Kim N. H. (2025). Managing dyslipidemia in chronic kidney disease: a comprehensive overview of evidence and recommendations. Korean J. Intern Med. 40, 876–889. 10.3904/kjim.2025.099 41223870 PMC12611488

[B20] Liaigre L. Guigui A. Manceau M. Cracowski J. L. Khouri C. Roustit M. (2025). Trial-level surrogacy of non-high-density and low-density lipoprotein cholesterol reduction on the clinical efficacy of statins. Eur. Heart J. Cardiovasc Pharmacother. 11, 387–392. 10.1093/ehjcvp/pvaf016 39999862 PMC12231127

[B21] Ma S. Qiu Y. Zhang C. (2024). Cytoskeleton rearrangement in podocytopathies: an update. Int. J. Mol. Sci. 25, 647. 10.3390/ijms25010647 38203817 PMC10779434

[B22] Meeuwisse C. Morgan C. J. Samuel S. Alexander R. T. Rodriguez-Lopez S. (2022). Rituximab use for the treatment of childhood nephrotic syndrome by Canadian pediatric nephrologists: a national survey. Can. J. Kidney Health Dis. 9, 20543581221079959. 10.1177/20543581221079959 35300066 PMC8922210

[B23] Naik V. Darandale A. Pal P. (2026). Emerging role of SGLT2 inhibitors beyond glycemic control: “cardiovascular and Renal Benefits and Risks”. Cardiovasc. Dis. Prev. Control 1, 27–36. 10.64229/v3v43h95

[B24] Qadri A. H. Prajapati J. Praghna D. Sinha A. Pasupulati A. K. (2025). Structural and functional insights of the podocyte slit diaphragm complex. Tissue Barriers 14, 2575198. 10.1080/21688370.2025.2575198 41100809 PMC13228966

[B25] Qi Y. Cao X. Gao X. Q. Cheng X. Fang Y. X. Zhang X. X. (2026). Long-term real-world outcomes of rituximab in primary membranous nephropathy: a 154-patient cohort study from China. Ren. Fail 48, 2612448. 10.1080/0886022x.2025.2612448 41582001 PMC12833891

[B26] Ravani P. Bonanni A. Rossi R. Caridi G. Ghiggeri G. M. (2016). Anti-CD20 antibodies for idiopathic nephrotic syndrome in children. Clin. J. Am. Soc. Nephrol. 11, 710–720. 10.2215/cjn.08500815 26585985 PMC4822672

[B27] Ruggenenti P. Ruggiero B. Cravedi P. Vivarelli M. Massella L. Marasà M. (2014). Rituximab in steroid-dependent or frequently relapsing idiopathic nephrotic syndrome. J. Am. Soc. Nephrol. 25, 850–863. 10.1681/asn.2013030251 24480824 PMC3968490

[B28] Sadeghi M. Dehnavi S. Shahbaz S. K. Koushki K. Butler A. E. Jamialahmadi T. (2025). Statins and adhesion molecules: a review of a novel pleiotropic property of statins. Immunol. Res. 73, 97. 10.1007/s12026-025-09653-2 40526251

[B29] Spampinato S. Tanderup K. Barcellini A. Burchardt E. Eminowicz G. Šegedin B. (2025). Impact of the common terminology criteria for adverse events (CTCAE) evolution on toxicity scoring in gynaecological radiotherapy. Radiother. Oncol. 207, 110881. 10.1016/j.radonc.2025.110881 40220903

[B30] Takahashi Y. Komine-Aizawa S. Takada K. Kanemaru K. Takahashi S. Hayakawa S. (2024). Anti-CD20 rituximab prevents Interleukin-13-Induced cytoskeletal remodeling of human podocytes Via off-target effects. J. Nihon Univ. Med. Assoc. 83, 135–141. 10.4264/numa.83.4_135

[B31] Tolerico M. Molina J. Insenga A. Carrazco A. Njeim R. Sloan A. (2025). Modulation of the apolipoprotein M/S1PR4 pathway reduces podocyte lipid overload in Alport syndrome via distinct autophagy and efflux mechanisms. J. Am. Soc. Nephrol. 37, 1457–1472. 10.1681/asn.0000000996 41563385 PMC13078258

[B32] Vargas-Brochero M. J. Lafaut E. Radhakrishnan Y. Russo I. Sethi S. Zand L. (2025). Long-term outcome of adult patients with membranous nephropathy treated with rituximab. Kidney Int. Rep. 10, 2630–2641. 10.1016/j.ekir.2025.05.013 40814621 PMC12348103

[B33] Wendt R. Sobhani A. Diefenhardt P. Trappe M. Völker L. A. (2024). An updated comprehensive review on diseases associated with nephrotic syndromes. Biomedicines 12, 2259. 10.3390/biomedicines12102259 39457572 PMC11504437

[B34] Wilson P. W. F. Polonsky T. S. Miedema M. D. Khera A. Kosinski A. S. Kuvin J. T. (2019). Systematic review for the 2018 AHA/ACC/AACVPR/AAPA/ABC/ACPM/ADA/AGS/APhA/ASPC/NLA/PCNA guideline on the management of blood cholesterol: a report of the American college of cardiology/american heart association task force on clinical practice guidelines. J. Am. Coll. Cardiol. 73, 3210–3227. 10.1016/j.jacc.2018.11.004 30423394

[B35] Xu J. Ding Y. Wan L. Yang Q. Qu Z. (2022). A prospective cohort study of rituximab in the treatment of refractory nephrotic syndrome. Int. Urol. Nephrol. 54, 121–130. 10.1007/s11255-021-02860-4 33905043 PMC8732927

[B36] Yu Z. Huang M. Qin Y. Li X. Zhao Y. Wang Y. (2025). Rituximab-associated adverse events in nephrotic syndrome: a systematic review and meta-analysis. Heliyon 11, e41212. 10.1016/j.heliyon.2024.e41212 39834424 PMC11745799

[B37] Zhao Y. X. Li X. Cheng X. Pan Y. Liu L. (2025). Efficacy and safety of rituximab in treating adult patients with minimal change disease and focal segmental glomerulosclerosis: a prospective study compared with glucocorticoids. Drug Des. Devel Ther. 19, 10571–10587. 10.2147/dddt.S549834 41334365 PMC12667706

[B38] Zhong H. Li H. Y. Zhou T. Weng W. (2023). Rituximab therapy in adults with steroid-dependent nephrotic syndrome. Arch. Med. Sci. 19, 577–585. 10.5114/aoms.2019.88404 37313189 PMC10259397

[B39] Zhu D. Judge P. K. Wanner C. Haynes R. Herrington W. G. (2025). The prevention and management of chronic kidney disease among patients with metabolic syndrome. Kidney Int. 107, 816–824. 10.1016/j.kint.2024.12.021 39986466

